# Idarucizumab Reversal in Subdural Hemorrhage: A Single-Center Experience

**DOI:** 10.3390/life15101617

**Published:** 2025-10-16

**Authors:** Anita Mrvar Brečko, Monika Simerl Jožef, Ana Trebše, Matija Zupan, Tomaž Velnar, Senta Frol

**Affiliations:** 1 Department of Anesthesiology and Surgical Intensive Care, University Medical Centre Ljubljana, 1000 Ljubljana, Slovenia; anitambrecko@gmail.com; 2 Department of Vascular Neurology, University Medical Centre Ljubljana, 1000 Ljubljana, Slovenia; monika.simerl@kclj.si (M.S.J.); ana.trebse@kclj.si (A.T.); matija.zupan@kclj.si (M.Z.); 3 Faculty of Medicine, University of Ljubljana, 1000 Ljubljana, Slovenia; 4 Department of Neurosurgery, University Medical Centre Ljubljana, 1000 Ljubljana, Slovenia; tomaz.velnar@kclj.si

**Keywords:** subdural hematoma, idarucizumab, dabigatran, anticoagulation reversal, case series

## Abstract

Prompt reversal of anticoagulation in the elderly population with subdural hematoma (SDH) is critical to reduce morbidity and facilitate timely surgical intervention. In patients receiving dabigatran, idarucizumab provides rapid anticoagulation reversal. We evaluated clinical and radiological outcomes of dabigatran-treated SDH patients receiving idarucizumab, including those undergoing surgical management. We conducted a single-center retrospective observational study of dabigatran-treated patients who received idarucizumab reversal for traumatic or spontaneous SDH between 2016 and 2024. Hematoma evolution was monitored using follow-up computed tomography. Clinical and neurological outcomes were recorded. Of eleven included patients (mean age 80.8 ± 6.7 years; 36% female), falls were the primary cause (64%). SDH was chronic in 64% and acute in 36%, with associated traumatic lesions in 33%. Surgical evacuation was performed in 82% of cases. Anticoagulation was resumed in 27% of patients within 3–4 weeks post-discharge. The median Glasgow Outcome Scale Extended (GOSE) score was 5, indicating moderate disability. In-hospital mortality was 9.1%. Idarucizumab enabled rapid and safe dabigatran reversal in this high-risk elderly cohort, supporting both surgical and conservative SDH management. Functional outcomes were moderate and mortality was low, underscoring its clinical utility. Targeted reversal strategies remain essential, and further research should refine long-term anticoagulation management.

## 1. Introduction

Direct oral anticoagulants (DOACs) have become the preferred agents for stroke prevention and systemic embolism management in patients with non-valvular atrial fibrillation (AF), as well as for the treatment of venous thromboembolism [1,2,3,4,5,6,7]. Their global uptake has expanded substantially in recent years. With increasing longevity, the prevalence of AF continues to rise, reaching up to 18% in individuals over 85 years of age [8,9,10,11]. Although DOACs demonstrate robust efficacy and a more favorable safety profile—particularly with respect to intracranial hemorrhage (ICH)—compared with vitamin K antagonists (VKAs) [12,13,14], hemorrhagic complications such as subdural hemorrhage (SDH) may still occur in a minority of patients [15,16].

In the elderly, the widespread use of DOACs, combined with frailty and a higher risk of falls, has contributed to a growing incidence of SDH [15,16,17]. Additional predisposing factors include cerebral atrophy, which enlarges the subdural space and increases strain on bridging cortical veins [18]. SDH is associated with considerable morbidity and mortality, with outcomes being particularly poor in male patients [19]. In anticoagulated individuals—whether on DOACs or VKAs—mortality following SDH may reach approximately 30% [20].

In the case of hemorrhagic complication in patients receiving DOACs, discontinuation of the drug and application of an existing reversal drug are recommended according to the current guidelines [21]. Surgical evacuation remains the standard treatment for symptomatic SDH associated with elevated intracranial pressure to prevent secondary brain injury [22,23,24,25,26,27,28]. Crucially, reversal of anticoagulation prior to neurosurgical intervention is also essential to reduce the risk of perioperative hemorrhage and recurrence of SDH [21,26,29,30,31].

Preoperative management begins with an imaging study, usually a non-contrast CT scan, to assess hematoma thickness, midline shift, and the presence of septa or membranes [22]. In chronic SDH (CSDH), MRI may be considered to better characterize mixed densities or multilocular collections [25]. Optimization of medical status is essential. Correction of coagulopathy is crucial, especially in patients receiving anticoagulant or antiplatelet therapy [32,33]. Hemodynamic stabilization, electrolyte correction, and measures to reduce the increased intracranial pressure (e.g., osmotic agents) are initiated as required. Preoperative neurological assessment, often using the Glasgow Coma Scale (GCS) or Markwalder grading, helps with risk stratification and prognosis [34]. When planning anesthesia, the patient’s age, frailty, and concomitant diseases must be considered. In selected cases, especially in frail elderly patients, less invasive procedures under local anesthesia may be preferable [35].

The choice of surgical technique depends on the characteristics of the hematoma, the patient’s comorbidities, and the surgeon’s experience. Several surgical techniques are available, ranging from minimally invasive to extensive procedures. In twist drill craniotomy, a small drill hole (2 to 5 mm) is created with a twist drill, followed by the insertion of a catheter into the subdural space for passive or closed drainage. It is often performed under local anesthesia and is therefore suitable for patients with significant anesthesia risks [24]. However, the recurrence rates are higher compared to burr hole craniotomy. Burr hole craniotomy is the most widely used technique, usually involving one or two drill holes, removal of the hematoma, irrigation, and placement of a closed subdural drainage system. This approach offers a favorable balance between safety, efficacy, and recurrence prevention [35]. Double-drilled holes may allow for more complete evacuation of septated hematomas but increase operative time and invasiveness [32]. Craniotomy is indicated for organized, thick or recurrent hematomas, or for acute SDH (ASDH) with a significant mass effect. It allows for the removal of membranes and a more complete evacuation under direct vision. However, craniotomy carries a greater surgical risk, a longer recovery time, and a higher complication rate, especially in older patients [33]. Endoscopic evacuation performed through a small craniotomy offers better visualization of the subdural space while minimizing invasiveness. Initial studies suggest potential benefits in reducing recurrence and complications, although the availability of equipment and surgical expertise remain limiting factors [23].

Postoperatively, patients require close neurological monitoring, often in an intensive care unit. CT imaging within 24 h is standard to detect residual hematoma or complications [22]. Placement of a subdural drain for 24–48 h significantly reduces the recurrence rate, although prolonged drainage may increase the risk of infection [35]. Anticonvulsants may be considered in ASDH or cases with cortical irritation, although their role in CSDH remains controversial [22,33]. Functional recovery often requires multidisciplinary rehabilitation in which motor, cognitive, and language deficits are treated accordingly.

First to be introduced to the world among DOACs was dabigatran in 2010. In Slovenia, it was registered and available to use soon after in 2011. It is a direct thrombin inhibitor. In the following years, the DOACs repertoire expanded with direct factor Xa inhibitors rivaroxaban, apixaban, and edoxaban. Dabigatran is the second most prescribed DOAC in the region, following apixaban. By 2015, nearly 19,000 persons in Slovenia were using DOACs [36], and the number today is probably higher.

Idarucizumab, a humanized monoclonal antibody fragment, binds to dabigatran with high affinity, thereby rapidly and specifically neutralizing its anticoagulant activity [37]. Growing real-world evidence indicates that idarucizumab use in patients with ICH, including SDH, is associated with low mortality and a low incidence of thromboembolic complications [37,38]. Accordingly, current guidelines recommend idarucizumab as the standard reversal agent for dabigatran-treated patients presenting with acute ICH [21,26,29,30,31,37]. While its safety and effectiveness has been well-established in acute ICH, specific evidence regarding its use in SDH is limited to a few existing studies and case reports [39,40,41,42,43,44].

Other reversal agents that are approved for use include target factor Xa, andexanet alfa, and 4-factor prothrombin complex concentrate (4F-PCC). These drugs are associated with similar effectiveness while carrying a higher risk of thrombotic complications compared to idarucizumab [45,46]. This retrospective, single-center study aims to assess the clinical outcomes of dabigatran-treated patients with traumatic and non-traumatic SDH following anticoagulation reversal with idarucizumab. To our knowledge, this is the first study to include non-traumatic SDH cases.

## 2. Materials and Methods

This retrospective observational case series included all consecutive patients admitted to the University Medical Centre (UMC) Ljubljana between September 2016 and July 2024. We included patients treated with dabigatran daily who presented with SDH regardless of concomitant trauma and received idarucizumab for anticoagulation reversal. All of the included patients were over 65 years old.

UMC Ljubljana is the national referral center for traumatic and non-traumatic brain injury in Slovenia. Patient management followed national guidelines, which emphasize rapid, interdisciplinary decision-making in emergencies. According to national protocols, anticoagulation is discontinued and reversed in patients with traumatic or non-traumatic brain injury, with individualized risk–benefit assessment. In all cases, dabigatran therapy was discontinued and 5 g of idarucizumab was administered intravenously immediately within minutes of SDH diagnosis confirmation on imaging. The relatively high cost of the drug (approximately EUR 2500 per dose) is fully reimbursed by the Health Insurance Institute of Slovenia (ZZZS), ensuring that cost considerations did not limit its use in eligible patients.

This study was approved by the Slovenian National Medical Ethics Committee, and written informed consent was obtained from all participants.

### 2.1. Clinical and Imaging Evaluation

Patients underwent initial clinical assessment on admission and were re-evaluated at discharge. Interim neurological deterioration during hospitalization was recorded. The mechanism of injury (if present), comorbidities, and treatment modality (surgical vs. conservative) were documented.

Initial diagnostic work-up included head computed tomography (CT) to classify the type and severity of SDH (ASDH, CSDH, or mixed) and to detect associated traumatic lesions (e.g., cerebral contusions, traumatic subarachnoid hemorrhage, and cerebral edema).

According to in-hospital practice and international guidelines, either surgical treatment or conservative management was performed.

Follow-up CT was obtained within 24 h after surgery or within several days in conservatively treated patients.

Anticoagulation was resumed in patients with complete resorption of the SDH and a high thromboembolic risk (high CHA_2_DS_2_-VASc score), provided they had a good functional status (mRS ≤ 3), no significant cognitive impairment (Mini Mental State Examination > 23), and no severe renal or hepatic dysfunction, thrombocytopenia, or anemia.

At least two years after hospitalization, the patients were followed-up via telephone (when contact information was available) or available electronic records to assess vital status, functional outcome using mRS, and anticoagulation resumption.

### 2.2. Variables Collected

The following patients’ parameters were systematically collected and analyzed by a team comprising an anesthesiologist, a neurosurgeon, and a neurologist (Table 1):

- **Demographic and clinical data:** Age, sex, injury mechanism, comorbidities.
- **Neurological assessment:** National Institutes of Health Stroke Scale (NIHSS) at admission and discharge, neurological examination including pupillary abnormalities, GCS at admission, pre-morbid modified Rankin Scale (mRS), and Glasgow Outcome Scale Extended (GOSE) score at discharge.
- **Imaging findings:** CT characteristics at admission and changes in follow-up imaging.
- **Anticoagulation and treatment:** Details of dabigatran therapy, timing of idarucizumab administration, resumption of anticoagulation, and surgical procedures.
- **Complications:** Postoperative complications, acute neurological deterioration during hospitalization, and ischemic and hemorrhagic events.

### 2.3. Statistical Analysis

Continuous variables are reported as mean ± standard deviation (SD) in normal distribution variables or median with interquartile range (IQR) in abnormal distribution. Categorical variables are presented as frequencies and relative frequencies. Simple statistical analysis was performed using Microsoft Excel 365.

## 3. Results

Eleven dabigatran-treated patients with traumatic or spontaneous SDH received idarucizumab. Patients’ parameters are presented in Table 1. The mean age was 80.8 ± 6.8 years, and 36% were female. Falls represented the most frequent mechanism of injury (64%). None of the patients were on concomitant antiplatelet therapy.

On admission, the median GCS score was 15 (range, 3–15), indicating that most patients were fully conscious, while some presented with severe impairment. Neurological dysfunction was common. A total of 73% of patients presented with some neurological deficit, with four patients (36%) presenting with anisocoria and four (36%) with motor deficits. Head CT demonstrated CSDH in 64% of cases, while ASDH and associated traumatic injuries (contusions, basal cistern compression) were also observed. Surgical intervention was required in 82% of patients. Craniotomy with surgical evacuation was performed in 55% of patients, one of which required additional external ventricular drainage. Two patients received intracranial pressure probe, and decompressive craniotomy was only performed in one patient.

No patients suffered from ischemic complications after the application of idarucizumab or postoperatively.

The overall in-hospital mortality rate was 9.1% (n = 1). The patient that did not survive was a 74-year-old male with multiple comorbidities, including esophageal carcinoma, who presented with a GCS score of 14, left-sided paresis, and anisocoria. He had a recent fall history. Head CT confirmed the diagnosis of ASDH, and surgical evacuation was required. Postoperatively, he suffered from a hematoma enlargement which caused an unsalvageable deterioration in his clinical status.

**Figure 1:**
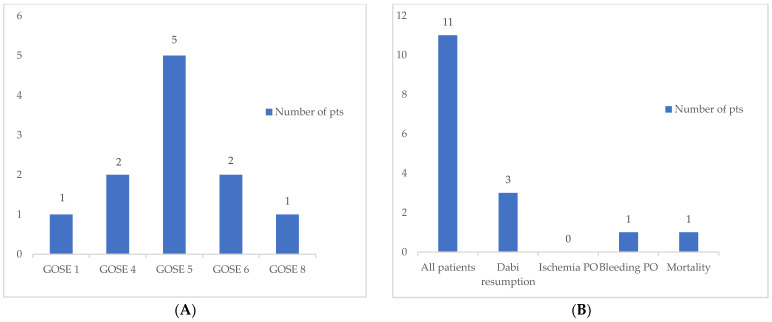
(**A**). Distribution of Glasgow Outcome Scale Extended Score. (**B**) Key outcomes of patients. Dabi: dabigatran; GOSE: Glasgow Outcome Scale Extended; PO: postoperatively; pts: patients.

The median GOSE score at discharge was 5, corresponding to moderate disability, with scores ranging from 4 (moderate disability) to 8 (good recovery). Patients with multiple comorbidities generally had poorer outcomes. Key clinical and demographic characteristics are summarized in Table 2 and Figure 1.

Anticoagulation with dabigatran was resumed in 27% of patients within 3–4 weeks after discharge. Subsequent reinitiation of anticoagulant therapy was not implemented for the remaining patients.

At a minimum follow-up of two years (Table 1), three patients (27%) were still alive. All three had resumed dabigatran at a lower dosage and experienced no new hemorrhagic or thrombotic events. Among them, two patients had a favorable outcome with a low mRS (≤1), while one patient had a poor outcome with a high mRS (5).

## 4. Discussion

This study provides further evidence supporting the safety and efficacy of idarucizumab for the reversal of the anticoagulant effect of dabigatran in patients with SDH requiring either surgical or conservative management. The low observed mortality rate (9.1%) aligns with prior findings and demonstrates idarucizumab’s capacity to mitigate perioperative hemorrhagic complications [37,38,39].

The mean age of the patients included in this cohort was 80.8 years, which is consistent with the age at which SDH occurs and use of DOACs is prevalent [47]. The age of our subjects is similar to other case reports and case series reporting idarucizumab use in dabigatran-treated patients with SDH [39,40,41,42,43,44].

The prevalence of CSDH (64%) is consistent with its etiology in elderly patients, in whom minor trauma and anticoagulation are major contributors. The predominance of falls (64%) underscores the importance of targeted fall-prevention strategies in this population. We also included cases of spontaneous SDH, of which we found no other cases reported for this age group.

The absence of significant thrombotic complications following idarucizumab administration highlights its favorable safety profile. One retrospective observational study from Japan observed ischemic complications in 13% of participants, which is more than in our case series [39]. The RE-VERSE AD Clinical Trial demonstrated rates of thrombotic complications in the range of 6.3–7.4% [37]. Because of low number of included cases in both our study and the Japanese study, generalizations cannot be made; however, it seems that the risk of ischemic complications is low. These findings reinforce current guideline recommendations for dabigatran reversal in acute ICH cases [21,26,29,30,31].

In our case series, one patient had hematoma expansion after the application of idarucizumab and suffered an unsalvageable decline, leading to death. Our rate of hemorrhagic complications was low (9.1%). Early application of idarucizumab, before aggravation, can reduce the rate of aggravation to the level seen in cases not administered DOACs or other anticoagulants, as suggested by one study [39]. In our study, the rate was even lower. The rate of hemorrhagic complications in DOAC patients can reach up to 30% [15].

However, the optimal timing of anticoagulation resumption remains unresolved and requires individualized decision-making that balances thromboembolic and hemorrhagic risks. Current evidence suggests that waiting at least 4 weeks before resuming anticoagulation may lower the risk of rebleeding, but this decision should always involve multidisciplinary input and close follow-up. The ischemic complications in the Japanese study occurred in the 4-week period: in two cases one week and in one case four weeks after the application of idarucizumab, before dabigatran resumption. It can be stipulated that the ischemic events were not a consequence of idarucizumab but rather of the absence of anticoagulation protection [39].

The reintroduction of anticoagulation after ICH due to trauma is complex and must be individualized. In mild trauma, anticoagulation may be considered after 4–8 weeks depending on hematoma resolution, thromboembolic risk, and neurological recovery. In moderate trauma, a delay of at least 8–12 weeks is generally advised, and resumption should only occur if the anticipated benefits outweigh the risks (e.g., patients with mechanical heart valves, prior venous thromboembolism, or AF with CHA_2_DS_2_-VASc ≥ 4) [48,49].

Several guidelines provide direction despite the absence of universally accepted timelines: (I) the European Heart Rhythm Association (EHRA, 2021) recommends resuming anticoagulation between 4 and 8 weeks after ICH following multidisciplinary assessment; (II) the American Heart Association/American Stroke Association (AHA/ASA, 2014) suggests that waiting at least one week may be reasonable, though optimal timing remains unclear [50,51]. The ongoing RESTART TICrH trial is expected to provide further evidence on the optimal timing for restarting DOACs after traumatic ICH [52].

Reported mortality in dabigatran-treated patients who did not receive reversal therapy varies considerably, depending on several factors, including etiology, injury severity, and patient age. In one retrospective study of five dabigatran-treated patients with closed head injury from ground-level falls, mortality reached 40% (2/5), compared with 0% among 15 warfarin-treated patients; however, the small sample size limits generalizability [53]. A larger analysis reported mortality of 12.5% in dabigatran-treated trauma patients versus 9.1% in controls without anticoagulation, a nonsignificant difference, suggesting that dabigatran itself may not substantially increase mortality in traumatic brain injury [54]. The RE-LY trial, which included both spontaneous and traumatic SDH, reported 21% overall mortality rate for dabigatran-treated patients [1,55].

The availability of idarucizumab likely contributes to improved survival in contemporary practice. In the RE-VERSE AD study, mortality among dabigatran-treated patients with ICH who received idarucizumab was 16.4%, which is lower than the rate reported in the RE-LY trial [37,55]. In our small cohort, we observed an even lower mortality of 9.1%, compared with 21% in the RE-LY trial and 16.4% in the RE-VERSE AD study [37,55].

Future directions should include large, multicenter studies to better characterize long-term outcomes and refine anticoagulation resumption protocols. In parallel, preventative interventions—particularly fall-prevention strategies—could meaningfully reduce the incidence of traumatic SDH in anticoagulated patients.

### Limitations

This study has several limitations that should be acknowledged. Its retrospective design introduces potential biases, such as selection bias and incomplete data capture, which may have influenced the findings. As our study was designed as a descriptive, hypothesis-generating case series with a small sample size, it was not statistically powered to perform meaningful subgroup or multivariable analyses that would enable the identification of predictors of clinical outcomes. This reduces the ability to detect rare events or to generalize the results to a wider population. In addition, the lack of long-term follow-up prevents firm conclusions about the sustained benefits of idarucizumab or the long-term safety of restarting anticoagulation. These limitations emphasize the need for larger, preferably multicenter cohort prospective studies with extended follow-up to confirm and build upon these findings.

## 5. Conclusions

Idarucizumab provides a rapid, effective, and safe means of reversing the anticoagulant effects of dabigatran in patients presenting with SDH. Its prompt action facilitates both timely surgical intervention and conservative management strategies, thereby mitigating the risk of ongoing or recurrent hemorrhage. This targeted reversal agent thus represents a pivotal tool in the acute care setting, where delays in management can significantly worsen neurological outcomes. Nevertheless, while current data supports its efficacy and safety in the acute phase, there remains an urgent need for larger, prospective studies to guide standardized, evidence-based protocols—particularly with respect to the optimal timing and strategy for resuming long-term anticoagulation in this high-risk, vulnerable patient population.

## Figures and Tables

**Table 1:** Patients’ parameters evaluated in the study.

Age	85	81	89	79	92	80	75	74	88	69	77
**Sex**	m	m	f	m	m	F	M	m	F	f	M
**Injury cause**	fall	fall	unknown	fall	fall	unknown	SE	fall	unknown	fall	Fall
**GCS**	15	15	12	15	15	3	SE	14	14	3	15
**Neuro. dysfunction at admission**	motor dysphasia, right-sided paresis	motor dysphasia, right-sided paresis	not possible due to the intubation	motor dysphasia, right-sided paresis	no symptoms	not possible due to the intubation	SE	left-sided paresis	no symptoms	GCS 3	no symptoms
**Pupils**	reactive, sym.	reactive, sym.	R > L	reactive, sym.	reactive, sym.	R > D	reactive, sym.	R > L	reactive, sym.	R > L	reactive, sym.
**CT**	CSDH	CSDH	ASDH	CSDH, compression of basal cisterns	CSDH	CSDH	CSDH	ASDH	CSDH	ASDH, Durret bleedings, traumatic SAH	ASDH, contusions
**D resumption**	No	No	No	No	yes	no	No	No	no	yes	yes
**Time to D resumption**	/	/	/	/	3 weeks	/	/	/	/	1 month	1 month
**Surgery**	yes	yes	ICP only	yes	no	DC	yes	yes	Yes, EVD	ICP only	No
**Ischemia PO**	no	no	no	no	no	no	no	no	no	no	No
**Bleeding PO**	no	no	no	no	no	no	no	HE	no	no	No
**Mortality**	no	no	no	no	no	no	no	yes	no	no	No
**GOSE**	5	5	6	5	4	4	5	1	5	6	8
**Concomitant diseases**	AF, PAOD	AF, AH	AF, AH	AF, AH, IS	AF, AH	AF, AH, IS, H	AF, AH	AF, AH, HF, esophageal cancer	AH	AF	AF, AH, H

m, male; f, female; GCS, Glasgow Coma Scale; D, dabigatran; PO, postoperatively; GOSE, Glasgow Outcome Scale Extended; sym, symmetrical; SE, status epilepticus; HE, hematoma enlargement; CT, computed tomography; CSDH, chronic subdural hematoma; ASDH, acute subdural hematoma; ICP, intracranial pressure probe; EVD, external ventricular drainage; DC, decompressive craniectomy; AF, atrial fibrillation; PAOD, peripheral arterial occlusive disease; AH, arterial hypertension; IS, ischemic stroke; H, hypercholesterolemia; HF, heart failure.

**Table 2:** The characteristics of the included patients.

Number of Patents	11
**Age**	Average age 80.8 years, range 69 to 92
**Sex**	64% male, 36% female
**Mechanism of injury**	Falls in 64%, the rest unknown
GCS *	Range from 3 to 15, median of 15
**Neurological dysfunction**	Various signs and symptoms
**Pupillary response**	Asymmetrical response in 36%
**CT findings**	CSDH in 64%, ASDH in 36%, concomitant traumatic findings like contusions and basal cistern compression in 27%
**Surgery**	Surgical intervention performed in 82% of cases
**Anticoagulant and antiplatelet use**	All patients used dabigatran only
**Dabigatran resumption**	27% resumed anticoagulation therapy post-discharge
**Antidote administration**	100%
**Mortality rate**	9.1%
GOSE *	Median GOSE 5, indicating moderate disability (range 4 to 8)
**Comorbid conditions**	Various
**2-year follow-up**	3 survivors (27%); mRS ≤ 1 in 2, mRS = 5 in 1; all on dabigatran 110 mg BID.

BID: twice daily; GCS, Glasgow Coma Scale; CT, computed tomography; CSDH, chronic subdural hematoma; ASDH, acute subdural hematoma; GOSE, Glasgow Outcome Scale Extended; * data was available for 10 out of 11 patients.

## Data Availability

The datasets generated and analyzed during the current study are not publicly available due to institutional privacy regulations, but are available from the corresponding author upon reasonable request.

## Abbreviations

The following abbreviations are used in this manuscript:

SDH	Subdural Hematoma
GOSE	Glasgow Outcome Scale Extended
DOACs	Direct Oral Anticoagulants
ICH	Intracerebral Hemorrhage
UMC	University Medical Center
CT	Computed Tomography
GCS	Glasgow Coma Scale
NIHSS	National Institutes of Health Stroke Scale
mRS	Modified Rankin Scale
SD	Standard deviation
IQR	Interquartile range
CSDH	Chronic Subdural Hematoma
ASDH	Acute Subdural Hematoma
AF	Atrial fibrillation
PAOD	Peripheral arterial occlusive disease
H	Hypercholesterolemia
HF	Heart failure
AH	Arterial hypertension
IS	Ischemic stroke
